# Role of the *WOR1* Promoter of Candida albicans in Opaque Commitment

**DOI:** 10.1128/mBio.02320-21

**Published:** 2021-09-07

**Authors:** Thomas P. Conway, Kayla Conway, Frank A. Boksa, Claude Pujol, Deborah Wessels, David R. Soll

**Affiliations:** a Developmental Studies Hybridoma Bank, Department of Biology, University of Iowa, Iowa City, Iowa, USA; Duke University

**Keywords:** *Candida albicans*, *WOR1* promoter, mass conversion, phenotypic commitment, phenotypic switching, white opaque switching

## Abstract

During induced differentiation, the process often involves a commitment event, after which induced cells, when returned to noninducing conditions, continue to differentiate. The commitment event is rarely identified. Candida albicans differentiates from the white to opaque phenotype, a prerequisite for mating and a process accompanying colonization of the lower gastrointestinal tract and skin. In analyses of white cell populations induced to synchronously differentiate from the white to opaque phenotype, opaque commitment occurs at approximately the same time as evagination and chitin ring formation in the process of daughter cell formation, several hours after the master switch gene *WOR1* is upregulated. Mutational analyses of transcription factor binding regions P1, P2, P3, P4, and P6 of the *WOR1* promoter reveal that individual deletion of any of the five transcription factor binding regions does not eliminate morphological differentiation to the opaque cell phenotype under opaque-inducing conditions, but individual deletion of P2, P3, or P4, blocks opaque commitment and maintenance of the opaque phenotype after transition to noninducing conditions. These results suggest that commitment occurs at the level of the *WOR1* promoter and that morphological differentiation can be dissociated from phenotypic commitment.

## INTRODUCTION

When a cell is induced to differentiate, there is a transition time to establish the new phenotype. Most differentiations involve changes in regulatory molecules during this period, which activate or suppress structural and physiological genes involved in generating the new phenotype. Since most studies of cellular differentiation involve comparisons between an initial phenotype and terminal phenotype, the transitional events that take place in establishing the new phenotype in many cases remain undescribed in space and time or, in the case of transient events, remain unidentified. In addition, attention is rarely paid to the commitment event, defined as the time at which a cell, induced to undergo a phenotypic transition, will continue to transition to the new phenotype when returned to noninducing conditions.

Candida albicans, an opportunistic fungal pathogen (1), undergoes a complex phenotypic transition from the white to the opaque phase that affects mating (2, 3), host colonization (4–9), biofilm formation (10), and gene expression (11–14). Like most inducible phenotypic transitions, little is known about the temporal events that occur in the transition from white to opaque or the nature of the opaque commitment event. However, there have been two notable studies in C. albicans directed at the reverse transition from opaque to white (15, 16), in which the time of the commitment event has been assessed. Here, we have employed an experimental protocol that has allowed us to assess the time at which white-phase cells, induced to differentiate to the opaque phase by culturing on *N*-acetylglucosamine agar in high CO_2_, commit to the opaque phase. In addition, we have analyzed the temporal relationship between opaque phase commitment, evagination, chitin ring formation at the mother cell-bud junction, and expression of the opaque-phase transcription activator Wor1. We show that commitment is dependent upon the presence of three transcription factor (TF) binding regions along the *WOR1* promoter. Finally, we demonstrate that cells lacking any one of three TF binding regions can express the opaque cell phenotype under inducing conditions, but cannot commit to the opaque phenotype, demonstrating that expression of the opaque phenotype can be dissociated from commitment to the opaque phenotype.

## RESULTS

### Strategy for assessing commitment.

**FIG 1:**
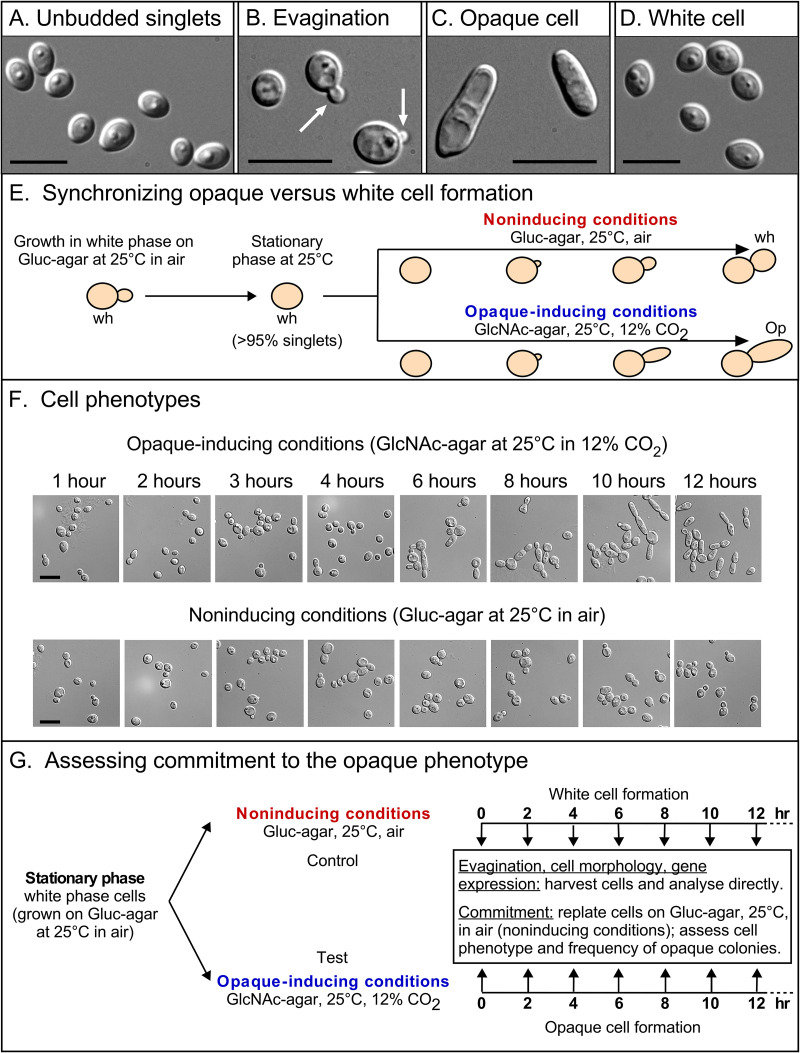
Strategy for assessing commitment in strain WO-1. (A) The unbudded yeast phase, white cell phenotype of the initial population prior to induction. (B) The evagination phenotype common to white and opaque phase cell populations. (C) Opaque cell phenotype. (D) White cell phenotype and daughter cells in control, noninduced cell populations. (E) The experimental procedure for initiating semisynchronous evagination and alternative daughter cell formation. (F) Cell phenotypes over time of cells plated under opaque-inducing conditions and noninducing conditions. (G) The sampling regimen for assessing evagination, cell morphology, gene expression, and commitment. Scale bars, 10 μm.

White-phase *MTL*-homozygous cells of strain WO-1 (*MTL*α/α) or P37005 (*MTL***a**/**a**) were grown to stationary phase at 25°C in air (0.04% CO_2_) on nutrient agar containing glucose as the carbon source (Gluc-agar). At stationary phase under these conditions, over 95% of cells accumulated in the unbudded white phase (Fig. 1A and B). To stimulate these cells to continue to multiply in the white phase (Fig. 1D), they were replated under the same conditions (Gluc-agar, 25°C, air; Fig. 1E). To induce the transition to the opaque phase (Fig. 1C), stationary-phase cells were replated on nutrient agar containing *N*-acetylglucosamine (GlcNAc) rather than glucose as the carbon source (GlcNAc-agar) and incubated at 25°C in 12% CO_2_ rather than in air (Fig. 1E). The conditions that maintained the white phenotype (i.e., did not induce opaque) were referred to as “noninducing” and the conditions that induced opaque cell formation were referred to as “opaque-inducing.” In Fig. 1F, images of cell morphologies over incubation time are presented for cells forming opaque cells under opaque-inducing conditions and cells forming white cells under noninducing conditions. To assess phenotypic commitment to the opaque phase, cells plated under opaque-inducing conditions were replated at time intervals over a 12-h period and assessed for opaque colony formation (Fig. 1G). As a control, parallel cultures of cells initially plated under noninducing conditions were replated under noninducing conditions and assessed for opaque colony formation (Fig. 1G). The proportion of cells that underwent commitment to the opaque phase under both inducing and noninducing conditions was measured as the proportion of cells in the final plating that formed opaque colonies (Fig. 1G). Since the results for strains WO-1 and P37005 were similar, the data for strain WO-1 is presented unless otherwise noted.

### Prerequisites for assessing commitment.

**FIG 2:**
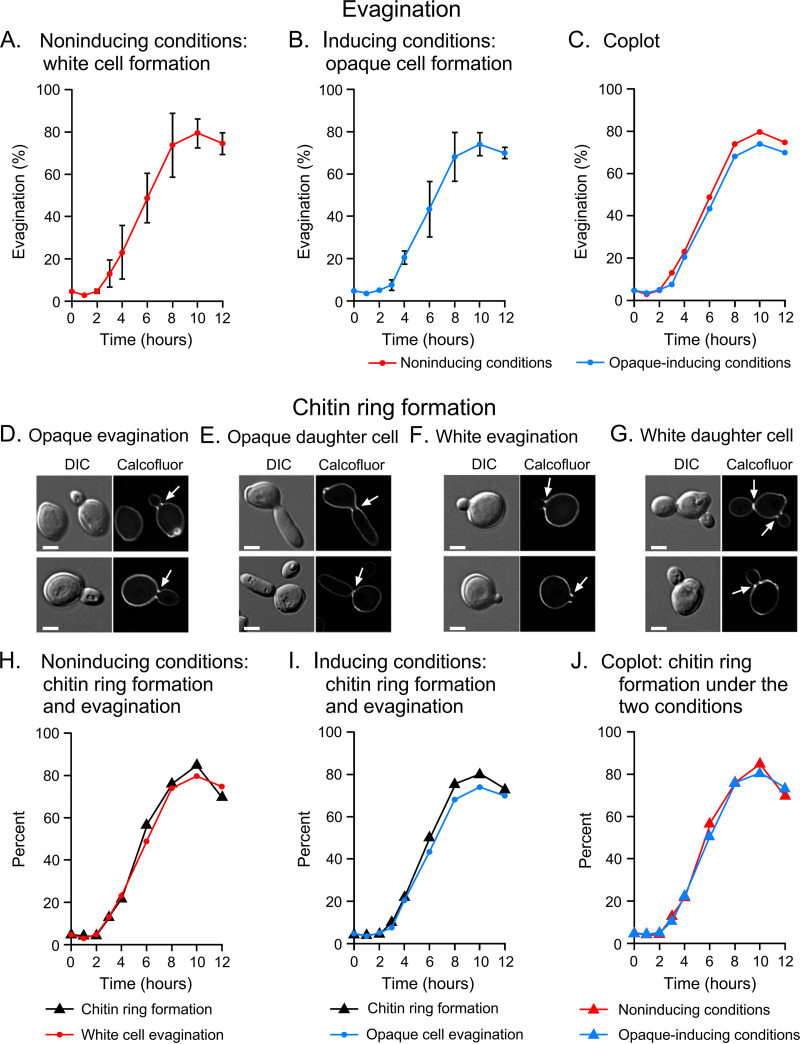
Evagination and filament ring formation in uninduced and opaque-induced cell populations of WO-1. (A to C) Kinetics of evagination in uninduced populations (A), opaque-induced populations (B), and coplots of the former two, respectively (C). Evaginated cells include cells with an evagination or daughter bud. (D to G) Calcofluor staining of the chitin rings at the constriction between mother cells and evaginations or mother cells and daughter cells. Scale bars, 2 μm. (H to J) Kinetics of chitin ring formation and evagination.

To assess opaque commitment in a population transitioning from white to opaque, the following five requisites were required: (i) phenotypic homogeneity of the initial stationary phase population of white cells; (ii) cellular changes that can be used to assess synchrony (e.g., evagination); (iii) an adequate degree of synchrony; (iv) an adequate degree of mass conversion to the opaque phenotype; and (v) temporal parallelism of alternative daughter cell formation between test (opaque) and control (white) populations. All of these requisites were achieved in the protocol outlined in Fig. 1E and G. As previously noted, growth to stationary phase of the initial white phase cell population on Gluc-agar at 25°C in air resulted in white-phase colonies containing over 95% white unbudded singlets, fulfilling the requisite for phenotypic homogeneity of the initial cell population. When these unbudded white-phase singlets were plated under both noninducing or opaque-inducing conditions, unbudded singlets began to evaginate in both cases at 3 h and continued to evaginate at a rate of 11% per hour for 5 h, attaining approximately 70% evagination by 8 h (Fig. 2A to C). In this case, “evaginated cells” were counted as mature cells with evaginations or maturing daughter cells. “Percent evagination” equaled the number of cells with incipient evaginations and maturing buds, divided by the total number of cells (unevaginated, with incipient evagination, and with maturing buds) times 100. The proportion of evaginated cells then plateaued, due to the maturation and release of unbudded daughter cells plus the return of evaginated mother cells to the unevaginated state (Fig. 2A to C). The kinetics of evagination were highly similar between uninduced and opaque-induced populations, as is evident in a coplot (Fig. 2C). Since the formation of a chitin ring at the mother cell-daughter cell junction had been shown to coincide with evagination of and commitment to the budding yeast phenotype (17–19), we examined uninduced and opaque-induced cell populations for the appearance of the chitin ring using calcofluor staining. Chitin rings localized to the junction of mother cells and evaginations of both uninduced and opaque-induced cell populations (Fig. 2D to G). The kinetics of chitin ring formation were highly similar to the kinetics of evagination in both populations (Fig. 2H and I) and highly similar between the alternative populations (Fig. 2J). These observations together demonstrated that the alternative populations fulfilled the requisites of synchrony and temporal parallelism.

### Commitment to the opaque phenotype.

**FIG 3:**
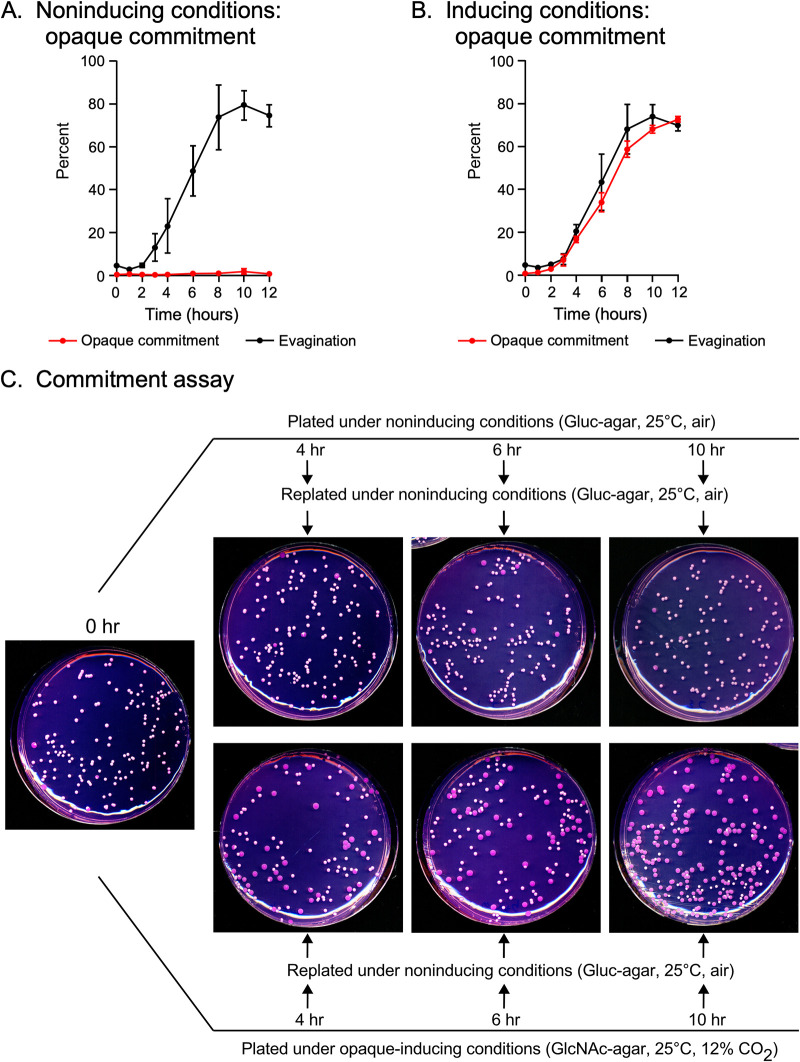
Commitment and evagination in WO-1. (A and B) Kinetics of commitment and evagination under noninducing (A) and opaque-inducing (B) conditions. (C) Commitment assay plates. To assess phenotypic commitment, cells plated at high density either on Gluc-agar at 25°C in air (noninducing conditions) or on GlcNAc-agar at 25°C in 12% CO_2_ (opaque-inducing conditions) were replated at low density under noninducing conditions. The agar contains phloxine B, which stains opaque colonies red or dark pink.

For control cultures initially plated under noninducing conditions (Gluc-agar, 25°C, air; Fig. 1G) and then replated at time intervals over a 12-h period, again under the same noninducing conditions, there was very low (close to 1%) to no formation of opaque colonies (Fig. 3A). However, when test cultures initially plated under opaque-inducing conditions (GlcNAc-agar, 25°C, 12% CO_2_; Fig. 1G) were replated under noninducing conditions (Gluc-agar, 25°C, air), an increase in opaque colony formation was observed, starting at 3 h (Fig. 3B). Over the following 5 h, opaque colony formation increased at approximately 10% per hour (Fig. 3B). The kinetics of commitment to the opaque phenotype (i.e., formation of opaque colonies after replating under noninducing conditions) was similar to the kinetics of evagination. However, the time at which 50% of cells had committed to the opaque phenotype for three independent experiments was consistently approximately 1 h later than the *T*_50_ of evagination and filament ring formation. Examples of dishes in the commitment assay are presented in Fig. 3C. It should be emphasized that this was true for both tested strains.

### Upregulation of *WOR1* precedes commitment.

**FIG 4:**
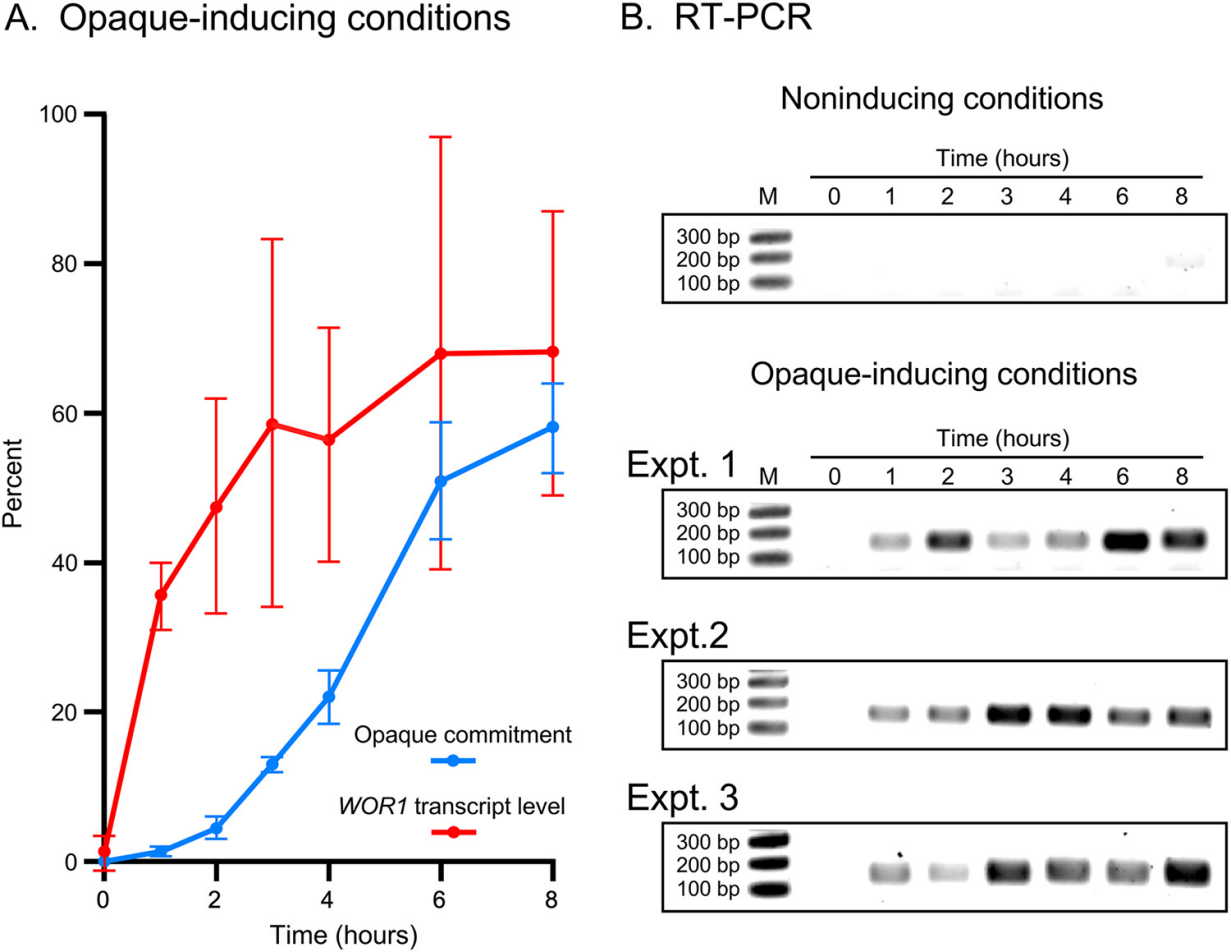
Expression of *WOR1* in relation to opaque commitment in WO-1. *WOR1* transcript levels were assessed by semiquantitative RT-PCR. (A) *WOR1* expression and commitment as a function of time under opaque-inducing conditions. Data are presented as the mean ± standard deviation for data from three separate experiments. Percent *WOR1* level is presented relative to the maximum level of pixels observed. (B) RT-PCR gel images under noninducing conditions and images of three experiments under opaque-inducing conditions.

*WOR1*, which encodes a key positive regulator of the white-to-opaque transition and is upregulated in opaque cells (20–22), was analyzed by semiquantitative reverse transcription-PCR (RT-PCR) under opaque-inducing conditions. An explanation for why we used direct RT-PCR measurements rather than measurements relative to a presumed constitutive transcript, such as that of *TDH3*, can be found in Fig. S1 in the supplemental material. Within the first hour of incubation, *WOR1* transcript levels increased to over 50% of the near maximum level attained at 6 h (Fig. 4A). The initial increase in the level of *WOR1* transcript, therefore, precluded evagination, chitin ring formation, and opaque commitment by several hours (Fig. 4A and B). Under noninducing conditions, there was no detectable increase in *WOR1* expression over the 8 h of analysis (Fig. 4B). It should again be emphasized that similar data were obtained for strain P37005.

10.1128/mBio.02320-21.3 FIG S1 Since the expression of housekeeping genes, commonly used as a reference to normalize qRT-PCR analyses, can change over time as unbudded white cells exit stationary phase under noninducing or opaque-induced conditions, they could not be used to normalize *WOR1* expression over time for qRT-PCR. Here, we have analyzed the expression of *TDH3*, a gene commonly used in qRT-PCR analyses of C. albicans, to demonstrate this point. Values are presented in threshold cycles (*C_T_*) in panel A and in RT-PCR gels in panel B. Because of such temporal changes and conditional variation in *TDH3* expression, and presumably other genes commonly used for data normalization, we have presented *WOR1* expression data using semiquantitative RT-PCR in Fig. 4, Fig. 7B, and Fig. 9. *C_T_* value, threshold cycle in PCR. Download FIG S1, PDF file, 0.3 MB. Copyright © 2021 Conway et al. 2021 Conway et al. https://creativecommons.org/licenses/by/4.0/ This content is distributed under the terms of the Creative Commons Attribution 4.0 International license.

### Deletion derivatives of the *WOR1* promoter.

**FIG 5:**
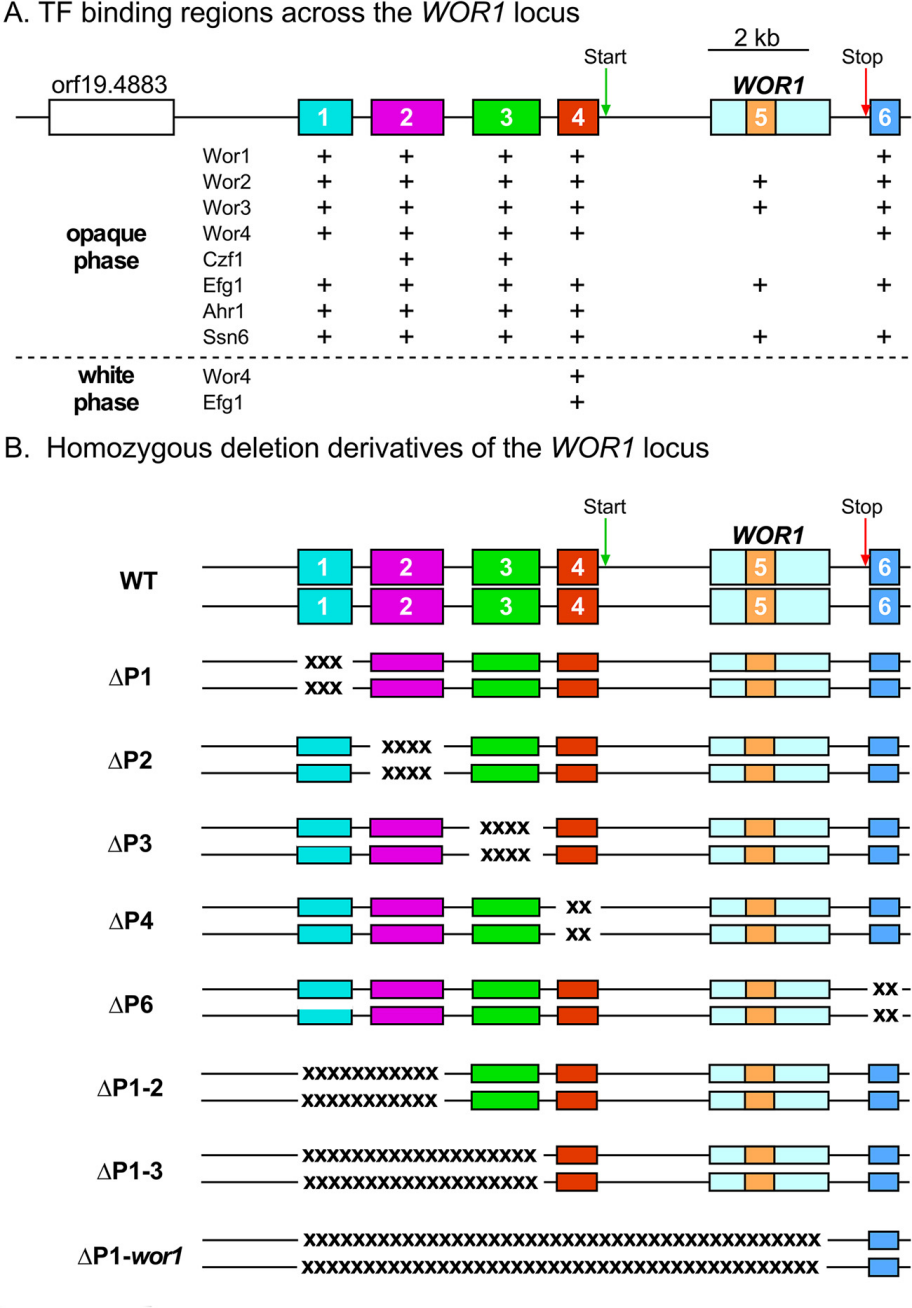
The homozygous deletion derivatives of the *WOR1* promoter generated in order to assess the role of transcription factor (TF) binding regions in the white-to-opaque transition. (A) Schematic of binding regions as identified by ChIP-chip analyses (24–26). (B) Homozygous deletion derivatives generated to assess the role of TF binding regions in switching and opaque commitment. XXXX-sequences represent deletions. TF binding regions are numbered in the wild-type (WT) promoter. Start, transcription start site; Stop, transcription stop site.

**FIG 6:**
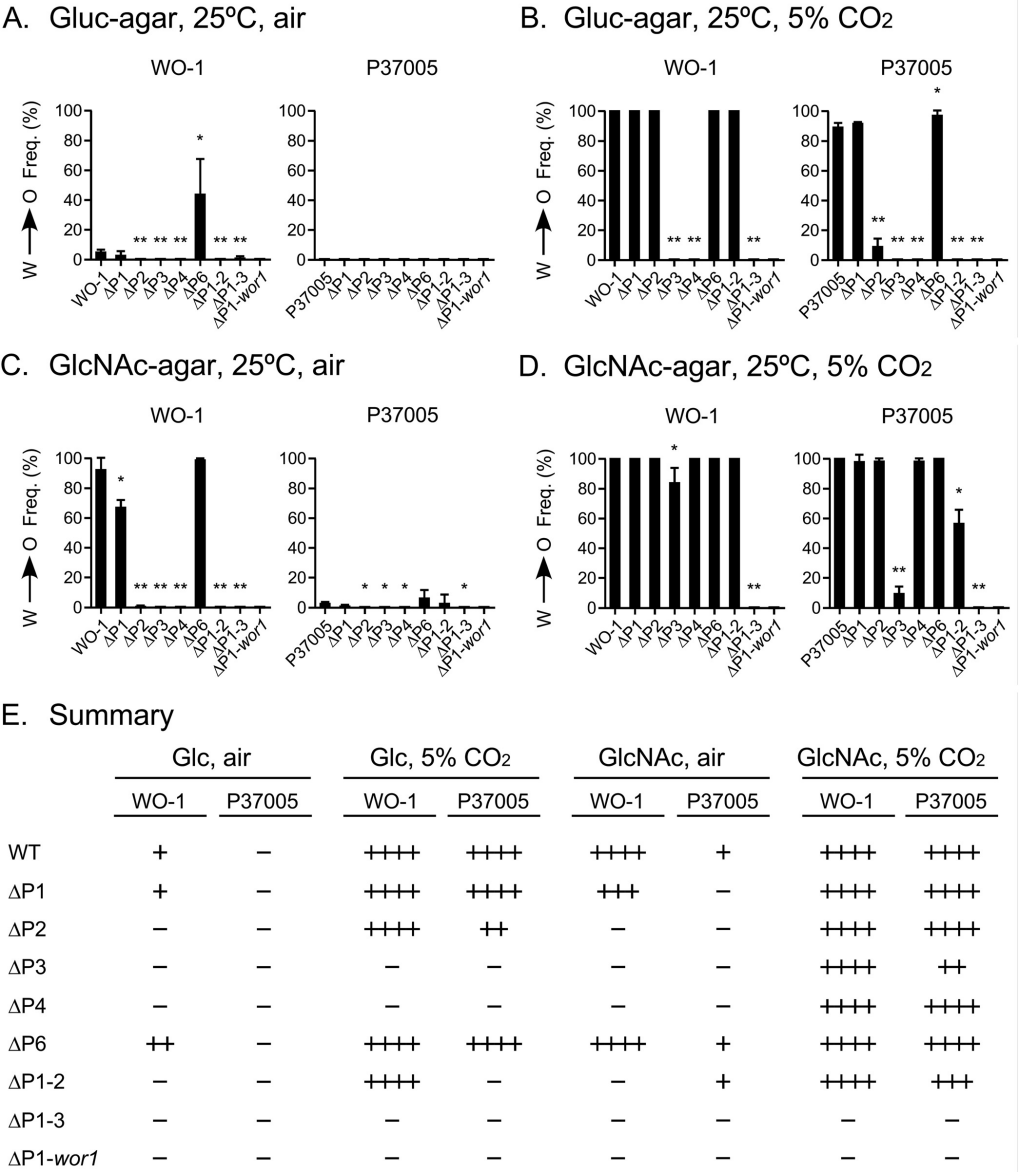
White-to-opaque switching by the WO-1 and P37005 deletion derivatives of the *WOR1* promoter under four sets of conditions. (A) Switching under noninducing conditions (Gluc-agar, 25°C, air). (B) Switching under high CO_2_-inducing conditions (Gluc-agar, 25°C, 5% CO_2_). (C) Switching under GlcNAc-inducing conditions (GlcNAc-agar, 25°C, air). (D) Switching under high CO_2_, GlcNAc-inducing conditions (GlcNAc-agar, 25°C, 5% CO_2_). Black bars, percent homogeneous opaque and opaque-sectored colonies. Close to 100% of colonies of strain P37005 ΔP3 contained a majority tiny elongate (gray) cells and a minority of white and opaque cells. Significant differences from WT were assessed by Student's *t* test: *, *P* < 0.05; **, *P* < 0.001. (E) Summary of switching results. −, 0%; +, 1 to 5%; ++, 6 to 50%; +++, 51 to 80%; ++++, 81 to 100%.

A number of transcription factors (TFs) have been shown to bind at four regions along the *WOR1* promoter (regions 1 through 4 in Fig. 5A), as well as a region in the open reading frame (region 5) and a region in the downstream 3′ sequence (region 6) (23–27) (Fig. 5A). Given the central role played by *WOR1* in the regulation of the white-to-opaque transition (20–22), the observation that the TFs coassemble in condensates that interact with the binding sites (28), and the role chromatin modification plays in epigenetic changes in phenotype (29–33), we considered the possibility that one or more of the TF binding sites played a role in commitment. To test this possibility, we generated homozygous deletion mutants for the individual binding regions 1, 2, 3, 4, and 6, and double and triple deletion mutants for multiple binding regions 1 and 2, and 1, 2, and 3, respectively, as well as a homozygous deletion of the entire *WOR1* locus, which included regions 1, 2, 3, and 4 on the promoter and the open reading frame containing region 5 (Fig. 5B). All of the binding site deletion mutants (ΔP1, ΔP2, ΔP3, ΔP4, ΔP6, ΔP1–2, ΔP1–3, and ΔP1-*wor1*, respectively) were generated in both the α/α strain WO-1 and the **a**/**a** strain P37005 (Fig. 5B) (3, 34). We first measured the frequency of white-to-opaque switching at 25°C under four sets of conditions: (i) spontaneous switching on Gluc-agar in air (noninducing); (ii) Gluc-agar in 5% CO2 (high CO2-inducing); (iii) GlcNAc-agar in air (GlcNAc-inducing); and (iv) GlcNAc-agar in 5% CO_2_ (GlcNAc/high CO_2_-inducing) (Fig. 6A to D, respectively). Under noninducing conditions, switching was low to undetectable in deletion mutants ΔP2, ΔP3, ΔP4, ΔP1–2, ΔP1–3, and ΔP1-*wor1* in strain WO-1 (Fig. 6A). However, switching occurred in ΔP1 at the same low frequency as wild type (WT) (3% and 5%, respectively), and in deletion mutant ΔP6, switching occurred at a frequency of 40% (Fig. 6A). In contrast, switching was undetectable in wild type and all eight deletion mutants of P37005 (Fig. 6A). Therefore, none of the individual binding regions functioned as *cis*-acting repressors of switching from white to opaque, with the exception of P6 and only in strain WO-1.

In preliminary experiments, we evaluated white-to-opaque switching under the same opaque-inducing conditions employed for commitment experiments in WO-1 (GlcNAc-agar, 12% CO_2_). These strong opaque-inducing conditions proved to be of limited use to assess switching differences between binding site mutants, since 100% of white stationary-phase cells of strains WO-1, ΔP1, ΔP2, ΔP3, ΔP4, and ΔP6 switched to opaque after 5 days of incubation. Milder opaque-inducing conditions were required for a finer analysis of the role of the binding sites in induced switching to opaque. Milder conditions were achieved by applying only one of the two inducing agents (i.e., high CO_2_ alone or GlcNAc alone) and reducing the CO_2_ levels from 12% to 5%.

Under high CO_2_-inducing conditions (Gluc-agar, 25°C, 5% CO_2_), wild type WO-1 and deletion mutants ΔP1, ΔP2, ΔP6, and ΔP1–2 underwent mass switching from white to opaque (Fig. 6B). In contrast, mutants ΔP3, ΔP4, and ΔP1–3 did not switch (Fig. 6B). Under the same high CO_2_-inducing conditions, the P37005 mutant derivatives responded in most cases like those of strain WO-1 with two exceptions: P37005 ΔP2 switched but at a lower frequency than WO-1 ΔP2, and ΔP1–2 did not switch (Fig. 6B). This suggests that P2 plays a weaker role in high CO_2_ activation of switching in strain WO-1 than in P37005. These results also suggest that P3 and P4 are essential for high CO_2_ activation of white-to-opaque switching.

Under GlcNAc-inducing conditions (GlcNAc-agar, 25°C, air), the ΔP2, ΔP3, ΔP4, ΔP1–2, and ΔP1–3 derivatives of WO-1 did not switch, whereas WO-1 WT, ΔP1, and ΔP6 exhibited high levels of switching (Fig. 6C). In contrast, neither WT nor any of the deletion derivatives of P37005 exhibited high levels of switching under GlcNAc-inducing conditions (Fig. 6C). In the case of the deletion derivative P37005 ΔP3, GlcNAc-inducing conditions resulted in a majority of gray cells (8, 35, 36), and a minority of white and opaque cells. These results indicated differences between strains in the role of the TFs in GlcNAc-induced switching.

Under GlcNAc/high CO_2_-inducing conditions, WT and all WO-1 promoter deletion derivatives except ΔP1–3 underwent mass switching to opaque (Fig. 6D), indicating that none of the five binding sites alone were individually essential for GlcNAc/high CO_2_ inducibility. However, simultaneous deletion of P1, P2, and P3 in the triple mutant ΔP1–3 abolished GlcNAc/high CO_2_ inducibility (Fig. 6D), indicating that the region encompassing P1 through P3 in WO-1 is essential. Under the same conditions, the switching responses of WT P37005 and the deletion derivatives were similar to those of WO-1 derivatives, except that switching to opaque was reduced in P37005 ΔP3 and ΔP1–2 (Fig. 6D). It should be noted that the preceding results presented in Fig. 6 are for stationary-phase unbudded white cells plated directly on agar under the four sets of conditions and colony phenotypes counted after 4 to 5 days without replating. It is not a measure of opaque commitment. The results of these experiments are synopsized in Fig. 6E.

### TF binding region deletions and commitment.

**FIG 7:**
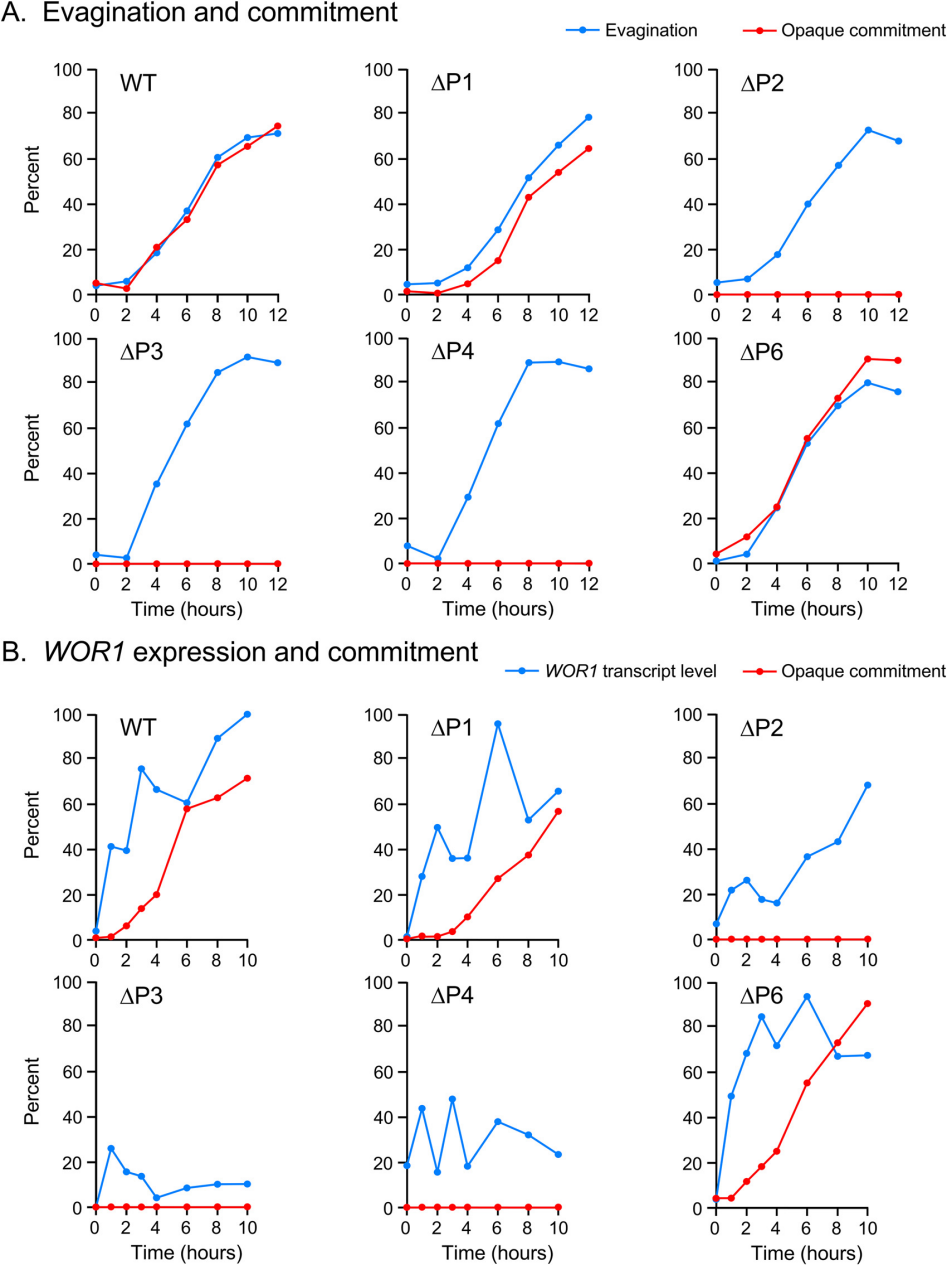
Evagination, commitment, and *WOR1* expression of the individual deletion derivatives (ΔP1, P2, ΔP3, ΔP4, and ΔP6) of the *WOR1* promoter induced to switch under opaque-inducing conditions (GlcNAc-agar, 25°C, 12% CO_2_). (A) Evagination and commitment. (B) Commitment and relative *WOR1* expression by RT-PCR.

After assessing their role in opaque induction, we tested the individual roles of the five TF binding regions, P1, P2, P3, P4, and P6, in opaque commitment using the protocol in Fig. 1. Stationary-phase, unbudded white cells of the parental wild-type strain WO-1 and the individual deletion strains ΔP1, ΔP2, ΔP3, ΔP4, and ΔP6 were first plated on GlcNAc-agar and incubated at 25°C in 12% CO_2_ (GlcNAc/high CO_2_ induction). WT, ΔP1, and ΔP6 cells underwent commitment with the same time kinetics as evagination, but the remaining three deletion derivatives (ΔP2, ΔP3, and ΔP4) did not undergo opaque commitment, even though all three evaginated with the same time kinetics as WT cells (Fig. 7A). These results suggested that while P1 and P6 were individually unessential for commitment, P2, P3, and P4 were individually essential.

### *WOR1* expression in deletion mutants.

Commitment and *WOR1* expression were compared between the parental WT strain WO-1 and the individual deletion derivatives. WO-1 WT, ΔP1, and ΔP6, the only strains that underwent commitment of the six compared strains, exhibited similarly high levels of *WOR1* expression (Fig. 7B). The three strains that did not undergo commitment, ΔP2, ΔP3, and ΔP4, all were induced to express *WOR1*, but at lower levels of approximately half that of WO-1 WT cells (Fig. 7B). These results suggest a correlation between the level of *WOR1* expression and commitment in populations induced to switch by GlcNAc/high CO_2_.

### Disassociation of opaque cell formation and commitment.

**FIG 8:**
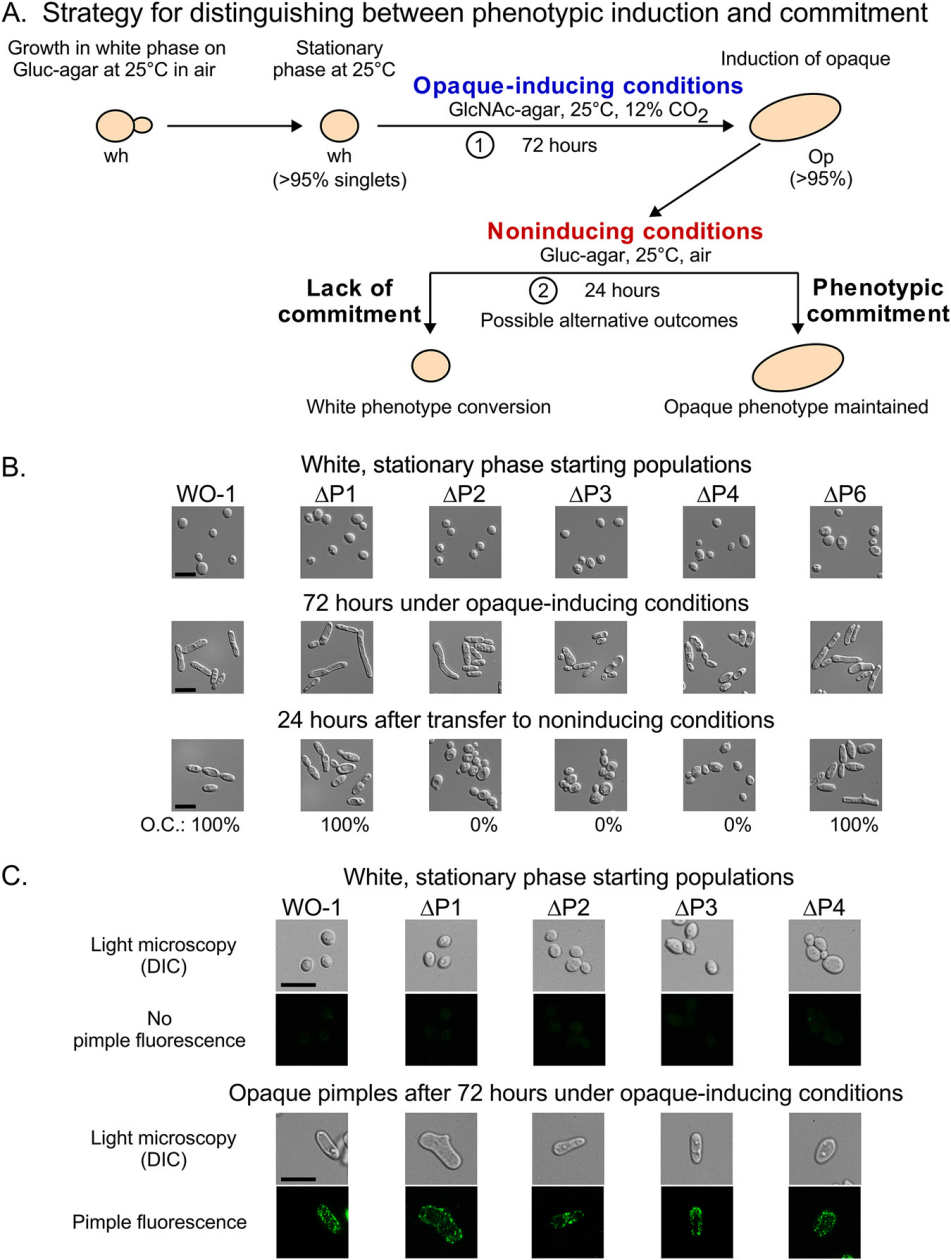
Phenotypic expression and commitment are dissociated in individual deletion derivatives ΔP2, ΔP3, and ΔP4, but not in ΔP1 and ΔP6. (A) The strategy for testing whether the individual deletion derivatives of P1, P2, P3, P4, and P6 can differentiate (i.e., express the opaque phenotype under inducing conditions) but not commit to the opaque phenotype. (B) Cellular phenotypes of initial stationary-phase white cultures, after 72 h incubation under opaque-inducing conditions (GlcNAc-agar, 25°C, 12% CO_2_), and after 24 subsequent hours of incubation under noninducing conditions (Gluc-agar, 25°C, air). (C) Opaque pimples on cells incubated for 72 h under opaque-inducing conditions. Cells were immunostained with anti-pimple polyclonal antibody. Scale bars, 10 μm.

Although the three WO-1 deletion mutants ΔP2, ΔP3, and ΔP4 did not commit to opaque cell formation, we observed that they readily formed opaque cells when white unbudded stationary-phase cells were plated and incubated on GlcNAc-agar at 25°C in both 5 and 12% CO_2_ (opaque-inducing conditions) (Fig. 6D and Fig. 8B). To verify whether opaque cells formed by ΔP2, ΔP3, and ΔP4 when incubated for an extended period of time under opaque-inducing conditions were not committed to the opaque phenotype, we performed the following experiment. White unbudded stationary-phase yeast cells of strains WO-1, ΔP1, ΔP2, ΔP3, ΔP4, and ΔP6 were incubated on GlcNAc-agar at 25°C in 12% CO_2_ (opaque-inducing conditions) for 72 h. These cells were then replated on Gluc-agar at 25°C in air (noninducing conditions), incubated for 24 h, and the colonies assessed for maintenance of the opaque phenotype (i.e., opaque commitment) (Fig. 8A). The initial preparations at 0 h, which were white cells that had been incubated under noninducing conditions, all formed white colonies containing 100% unbudded white-phase cells (Fig. 8B). When white cells of WO-1 and the deletion derivatives were replated under opaque-inducing conditions and then incubated for 72 h, the majority of colonies were opaque and contained opaque cells (Fig. 8B). To test that the cells were opaque, they were stained with anti-pimple polyclonal antibody (37, 38). The pAb stained the surface of opaque cells of all strains in a similar punctate fashion, demonstrating that they were all bona fide opaque cells (Fig. 8C). When opaque cells from the 72-h opaque colonies were replated under noninducing conditions and incubated for an additional 24 h (Fig. 8A), those from strains WO-1, ΔP1, and ΔP6, continued to grow exclusively in the opaque phase, indicating they had committed to the opaque phenotype. However, when opaque cells from ΔP2, ΔP3, and ΔP4 were replated, they formed microcolonies containing exclusively white cells (Fig. 8B), indicating that they had not committed to the opaque phenotype.

**FIG 9:**
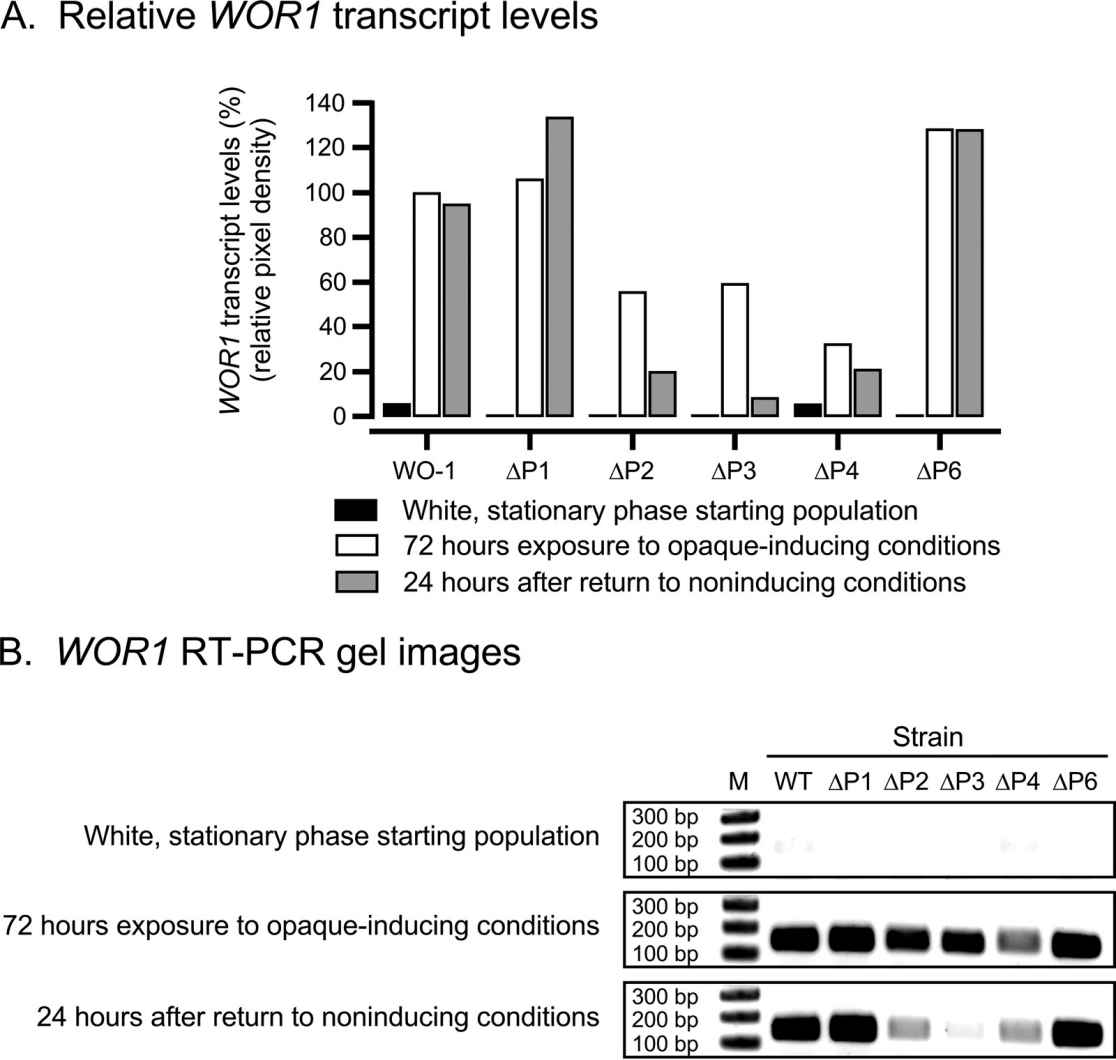
*WOR1* levels of promoter deletion derivatives are consistent with commitment or lack thereof. (A) Relative *WOR1* transcript levels of WO-1 WT, ΔP1, ΔP2, ΔP3, ΔP4, and ΔP6 derivatives for white stationary-phase cells at time zero, after 72 h under opaque-inducing conditions, and 24 h after the preceding 72-h cultures were replated under noninducing conditions. Levels are relative to expression of WT after 72 h under opaque-inducing conditions. (B) Gel images of RT-PCRs.

Since the capacity of the deletion derivatives to express the opaque phenotype is presumably mediated through *WOR1* expression, we measured the levels of *WOR1* expression in WO-1 WT, ΔP1, ΔP2, ΔP3, ΔP4, and ΔP6 by semiquantitative RT-PCR in the initial white cell preparations, after 72 h under opaque-inducing conditions, and then after 24 additional hours after replating under noninducing conditions (Fig. 9A and B). In the initial white stationary-phase cell populations, the levels of *WOR1* transcript for all strains were low to negligible (Fig. 9A and B). After 72 h under opaque-inducing conditions, the levels of *WOR1* expression in strains ΔP1 and ΔP6 were equal to or higher than that of WO-1 WT, respectively (Fig. 9A and B). In contrast, after 72 h under opaque-inducing conditions, the levels of *WOR1* expression in strains ΔP2, ΔP3, and ΔP4 were, respectively, reduced by 42%, 40%, and 70% relative to WO-1 WT. After 24 subsequent hours under noninducing conditions, the levels of expression in WT, ΔP1, and ΔP6 were similar to those under opaque-inducing conditions, but the levels of ΔP2, ΔP3, and ΔP4 were reduced to 20%, 8%, and 20% that of WO-1 WT, respectively (Fig. 9A and B). Together, these results suggest that cells can express the opaque phenotype without committing to the opaque phase, that P2, P3, and P4 are each individually essential for commitment to opaque but not to expressing the opaque phenotype, and that P2, P3, and P4 are each individually involved in the level of *WOR1* expression. In addition, these results suggest that cells may have to express a threshold level of *WOR1* in order to commit to the opaque phenotype.

## DISCUSSION

The transition of C. albicans and related species from the white to opaque phenotype is complex, affecting a large number of genes, cellular morphology, physiological characteristics, and host colonization (4, 5, 34, 39–47). Here, we have used an experimental protocol that allowed us to investigate how white cells induced to differentiate to the opaque phenotype will commit to that phenotype. By commitment, we mean the time after which opaque-induced white cells, when replated under noninducing conditions, continue to transition to and then maintain the opaque phenotype. The results show that opaque commitment occurs at the time induced cells evaginate and form a chitin ring at the mother cell-bud junction, several hours after upregulation of the TF *WOR1*, which encodes a transcription factor essential for switching. Functional analysis of deletion derivatives of the *WOR1* promoter further reveals a fundamental role of transcription factor binding sites along the *WOR1* promoter in the commitment event, and the dissociation of phenotypic expression and commitment involving TF binding sites.

Commitment to opaque in an opaque-induced population occurs with the same time kinetics as evagination and chitin ring formation, suggesting a relationship that may be causal. The formation of the chitin ring in a budding cell occurs at the mother cell-bud junction and in a hypha-forming cell along the hyphal tube, approximately two μm from the mother cell-tube junction. The chitin ring in both phenotypes forms over a cortical filament ring (48) composed in part of septins (49, 50), at the location of septum formation. Although commitment to the bud or hypha phenotype assessed by pH-regulated dimorphism (51) correlates with the timing of chitin ring formation (18, 19, 48), like commitment to opaque, there is no proof that chitin ring formation is causatively related to commitment. It seems more likely that commitment involves the interacting network of TFs regulating switching at the level of promoter function (23–27) and, in particular, the TF Wor1, the first TF implicated in the regulation of the white-to-opaque transition (20–22).

*WOR1* has been shown to be upregulated in opaque cells and, by mutational and overexpression analyses, to be essential for the transition from white to opaque. Deletion of *WOR1* in strains harboring functional copies of the white-specific TF gene *EFG1* are blocked in the white phase and cannot be induced to switch to opaque (9, 20–22). Using our experimental protocol, we found that *WOR1* was upregulated several hours prior to commitment, reaching 50% maximum expression after 1 h and near-maximum expression after 3 h of incubation under inducing conditions, the latter when only 15% of cells had committed to opaque. The possibility exists that this delay represents latency between mRNA production and the generation of threshold levels of functional protein. The 2-kb 5′ untranslated region (UTR) of the *WOR1* transcript has been shown to play a role by reducing translation efficiency (52) and could therefore potentially be involved in delayed commitment. That does not exclude the possibility that the 5′-UTR may be involved in the conformational changes in chromatin structure involved in upregulation or opaque commitment. Nevertheless, Lohse and Johnson demonstrated that decreased *WOR1* transcript levels correlated closely with protein levels during the temperature-induced opaque-to-white transition, suggesting that measured transcript levels in our experiments may reflect protein levels. Interestingly, during the temperature-induced transition from the opaque to white phenotype, *WOR1* mRNA levels were shown to substantially decrease before commitment (53). After 3 h, Wor1 protein levels decreased by more than 60% before any commitment to white had occurred (53). This suggests that similar mechanisms or reverse changes could be controlling commitment to the white or opaque state at the *WOR1* locus. Latency to opaque commitment may reflect a delay in establishing an epigenetic heritable state, just as latency to white commitment may reflect a delay in reversing establishment of the opaque epigenetic state. Although the induced expression of *WOR1* occurs several hours prior to commitment, it does not exclude it from involvement in commitment, since a change, either at the *WOR1* locus or at the level of translation or protein modification, may be time dependent, taking several hours to effect commitment.

Since TF binding regions regulate *WOR1* expression, we investigated whether they played a role in the commitment process by a functional analysis of deletion mutants of each of the TF binding regions in an α/α strain and an **a**/**a** strain. Using the opaque-inducing conditions of high CO_2_ (54) or GlcNAc as the sugar source (55), we found that the role of several of the essential binding regions differed between inducers and between strains. Two TF binding regions, P3 and P4, played essential roles in opaque induction by both inducers in both strains. However, a combination of the two inducers, high CO_2_ and GlcNAc, induced mass switching to opaque in all but one of the deletion mutants, P37005 ΔP3, although the combination did induce switching in this derivative, but at a lower frequency. When we tested whether induced opaque cells of the individual deletion mutants of the five TF binding regions of strain WO-1 were committed to the opaque phenotype, we found that while ΔP1 and ΔP6, like WO-1 wild type, were committed, the individual deletion mutants ΔP2, ΔP3, and ΔP4 were uncommitted, promptly reverting to the white state when transferred to noninducing conditions. The level of *WOR1* expression in opaque cells of ΔP1 and ΔP6 induced by high CO_2_ and GlcNAc was similar to that of WO-1 wild type cells induced by high CO_2_ and GlcNAc. The *WOR1* transcript levels of the uncommitted cells of mutants ΔP2, ΔP3, and ΔP4 were approximately half that of wild type. Consistent with that correlation, when wild type, ΔP1, and ΔP6 opaque cells were transferred from opaque-inducing to noninducing conditions, they continued to express the opaque cell morphology and express *WOR1* at levels similar to those observed under opaque-inducing conditions. However, when opaque cells of ΔP2, ΔP3, and ΔP4 were replated under noninducing conditions, they dedifferentiated to the white cell morphology and expressed *WOR1* at levels of 20%, 8%, and 20%, respectively, that of wild-type cells. These results indicate that the commitment event requires the promoter region spanning P2, P3, and P4, which includes base pairs −6536 to −2138 relative to the *WOR1* initiation codon. These regions do not appear to be associated with a common set of TFs regulating the white-to-opaque transition and controlling commitment, since P1 and P6 bind to most of the same TFs as the essential sites P3, P4, and P5 (Fig. 5A). In the opaque phase, the TF Czf1 binds only to P2 and P3 but doesn’t bind P4, and in the white phase, Wor4 and Efg1 only bind P4. Binding of Cfz1 to P2 and P3 in the opaque phase cannot explain our results since deletion of *CZF1* drastically reduces switching to opaque but does not eliminate it (23). In contrast, deletion of *WOR4* has been reported to block switching from white to opaque. It is nevertheless unlikely that the specific binding of Wor4 to P4 in the white phase could explain our results, since Wor4 binds to P1, P2, P3, P4, and P6 in the opaque phase. Recently, it has been shown that the white and opaque network TFs contain prion-like domains that function in the recruitment and formation of multifactorial TF aggregates that condensate on DNA (28). The assembly of such TF complexes on promoters has been suggested to play a regulatory role in the white-to-opaque transition (28). The possibility exists that formation of an activated TF complex over the P2-P3-P4 region is necessary for robust transcription at the *WOR1* locus, and that deletion of P2, P3, or P4 abrogates formation of such a TF complex.

In conclusion, our results indicate that induction of the white-to-opaque transition by *MTL*-homozygous strains of C. albicans, by either GlcNAc or high CO_2_, exhibits different dependences on the enhancer regions of the *WOR1* locus. Differences also exist between the two tested strains, one α/α and the other **a**/**a**. When the transition is induced by a combination of GlcNAc and high CO_2_, however, all but one of the tested individual deletion mutants and the double mutants in both strains are induced to undergo mass switching to opaque. However, when commitment was assessed in individual WO-1 mutants of the TF binding regions, the deletion mutants ΔP2, ΔP3, and ΔP4 did not undergo commitment, even though they did undergo the transition in phenotype. Thus, the phenotypic transition can be dissociated from the commitment event. One possible explanation may relate to the reduction in *WOR1* expression observed in the ΔP2, ΔP3, and ΔP4 mutants, accompanying the transition. Zordan et al. (20) and Srikantha et al. (22) presented evidence suggesting that the level of *WOR1* expression had to reach a threshold level to mediate a switch from white to opaque. In addition, ChIP-chip results indicate that Wor1 itself induces expression by binding to all five of the tested TF binding regions of the *WOR1* locus. Based on this concept, one possible explanation for dissociation is that the lower level of *WOR1* expression in ΔP2, ΔP3, and ΔP4 reaches the threshold for a phenotypic switch, but does not reach the higher *WOR1* threshold required for commitment. An alternative but possibly nonexclusive explanation is based on chromatin structure. The three regions, P2, P3, and P4, may be necessary for an epigenetic, heritable change in the tertiary structure of the *WOR1* promoter, basic to commitment. Heritable epigenetic changes, in many cases, have been demonstrated to be due to secondary modifications of histones and DNA methylations, which have been shown to be involved in stable changes in phenotype (56–59), including transitions to neoplasia, metastasis, and tumorigenesis (57, 59–61). In this regard, it is noteworthy that mutations in histone deacetylases have dramatic effects on the frequency of white-to-opaque and opaque-to-white switching (29–31). We therefore suggest the alternative hypothesis that P2, P3, and P4 are each required for a heritable epigenetic change in the tertiary structure of promoter chromatin, which is the basis of commitment to the opaque phenotype. Whichever of the alternative hypotheses proves correct, our results demonstrate that commitment to the opaque phenotype is associated with the P2, P3, and P4 regions of the *WOR1* promoter.

## MATERIALS AND METHODS

### Strains and media.

The C. albicans strains, genotypes, and sources used in this study are listed in Table S1 in the supplemental material. All strains were maintained at room temperature on nutrient agar containing either YPD medium (1% yeast extract, 2% peptone, 2% glucose) or supplemented Lee’s medium (62), with the latter containing either 1.25% glucose (Gluc-agar) or 2% *N*-acetylglucosamine (GlcNAc-agar). To distinguish opaque colonies and sectors, the vital stain phloxine B, which differentially stains opaque cells red (37), was added to Gluc- and GlcNAc-agar medium at a concentration of 5 μg/liter. Frozen stocks of all strains were stored at −80°C.

10.1128/mBio.02320-21.1 TABLE S1 Strains used in this study Table S1, PDF file, 0.09 MB. Copyright © 2021 Conway et al. 2021 Conway et al. https://creativecommons.org/licenses/by/4.0/ This content is distributed under the terms of the Creative Commons Attribution 4.0 International license.

### Evagination kinetics.

Cells released from stationary phase during opaque commitment assays were assessed at time intervals for the appearance of visible evaginations and daughter cells. Single cells with a visible evagination and mother cells with a primary daughter cell were included in a calculation of the percent evaginated cells in a sample. Cells with evaginations or unseparated daughter cells (buds) were considered a single evagination event. In summary, % evagination = ([evaginated mother cells + mother cells with a daughter cell]/[total number of unevaginated mother cells + evaginated mother cells + budded mother cells]) × 100.

### Switching and commitment assays.

White cells were grown on Gluc-agar at 25°C in air (“noninducing conditions”) for a minimum of 5 days to reach stationary phase. To assess the frequency of white-opaque switching, white cells were replated at approximately 200 cells/plate on Gluc-agar or GlcNAc-agar containing phloxine B and incubated at 25°C in air or in 5% CO_2_. After 5 days, the proportions of white and opaque colonies were measured (37). To assess opaque commitment, white cells were seeded at high density (10^6^ cells/plate) and incubated under noninducing conditions or on GlcNAc-agar at 25°C and 12% CO_2_ (“opaque-inducing conditions”). At specific time intervals, cells were resuspended in one ml of sterile distilled H_2_O, microscopically imaged for cell phenotype, and replated on Gluc-agar containing phloxine B and incubated at 25°C in air. Cultures were incubated for 5 days and scored for the proportion of opaque colonies.

### Staining of the chitin ring.

Cells were fixed with 2% paraformaldehyde in 1× phosphate-buffered saline (PBS), washed three times with sterile distilled H_2_O, and then stained with calcofluor (Fluorescent Brightener 28, Sigma-Aldrich, St. Louis, MO). Stained cells were imaged with a Leica TCS SP8 confocal microscope.

### Immunolocalization of the opaque-specific pimple marker.

Rabbit-derived polyclonal anti-pimple antiserum against an opaque pimple antigen (38) was used to visualize the formation of opaque-specific pimples by immunocytochemistry. Opaque cells were heat killed in a 70°C water bath for 1 h, pelleted, and resuspended in 1× PBS supplemented with 10% normal goat serum to block nonspecific binding. A 1:50 dilution of rabbit serum was preabsorbed five times with 10^6^ heat-killed WO-1 white cells to remove antibodies to surface antigens common to white and opaque cells (38). After staining with the primary antiserum, cells were washed with 1× PBS and treated with Alexa Fluor 488-tagged goat anti-rabbit secondary antibody (Jackson ImmunoResearch, West Grove, PA). Fluorescent images were captured using a Leica TCS SP8 confocal microscope, and all images were similarly processed with Image J software.

### Semiquantitative RT-PCR.

Cells were resuspended in RNAlater solution (Ambion, Life Technologies, Carlsbad, CA), incubated for 1 h at 4°C, and then stored at –80°C. Immediately prior to RNA extraction, cell samples were homogenized by mechanical lysis with acid-washed glass beads in a bead beater. DNA contamination was removed from samples by DNase treatment using the TURBO DNA-free kit (Ambion). The RNA samples were cleaned using the RNeasy minikit (Qiagen). The RNA content of each sample was quantified using a NanoDrop One spectrophotometer (Thermo Fisher Scientific, Wilmington, DE). All RT-PCRs performed for semiquantitative analyses were carried out using a Superscript III OneStep RT-PCR kit (Life Technologies, Carlsbad, CA) in accordance with the manufacturer’s instructions. The primers used for analysis of *TDH3* and *WOR1* transcript levels can be found in Table S2. For imaging and analysis, equal amounts of the reaction products were electrophoresed through a 1.6% agarose gel at 100 V for 20 min in an EP-2015 RunOne electrophoresis unit (Embi Tec, San Diego, CA). Gels were stained with SYBR Safe DNA gel stain (Life Technologies) and subsequently imaged with a LI-COR Odyssey Fc Dual Mode imaging system (LI-COR, Lincoln, NE). Quantification of band intensity (i.e., pixel density) was performed with Image Studio Software (LI-COR).

10.1128/mBio.02320-21.2 TABLE S2 Primers used in this study Table S2, PDF file, 0.04 MB. Copyright © 2021 Conway et al. 2021 Conway et al. https://creativecommons.org/licenses/by/4.0/ This content is distributed under the terms of the Creative Commons Attribution 4.0 International license.

To avoid saturation in our RT-PCRs and in pixel density analyses, we performed a reaction dilution series using the RNA from the time point sample that yielded the highest band intensity in the initial calibration reactions. The RNA concentrations assessed in our dilution series were 0.0625 ng, 0.09375 ng, 0.125 ng, 0.1875 ng, 0.375 ng, 0.5 ng, 0.75 ng, 1.0 ng, 1.5 ng, and 2.0 ng. Based on the results, we chose to carry out all RT-PCRs with 0.5 ng of RNA, since the resulting signal was within the linear range.

### Generation of deletion derivatives of the *WOR1* promoter.

Deletion derivatives of the *WOR1* locus were generated in strains WO-1 (α/α) and P37005 (**a**/**a**) (Table S1). The vector pSFS2, which contains a recyclable flipper cassette harboring a dominant nourseothricin resistance marker (ca*SAT1*) (63), was used to generate the TF binding region mutants. First, the 5′ and 3′ sequences flanking the TF binding regions were amplified by PCR from genomic DNA of C. albicans strain SC5314 (**a**/α) using the primer pairs listed in Table S2. The amplified 5′ and 3′ flanking regions and pSFS2 were digested with the corresponding restriction enzymes (Table S2). The digested flanking sequence fragments were then ligated into the digested vector using T4 ligase in order to obtain a plasmid containing the recyclable deletion cassette flanked by fragments corresponding to the endogenous 5′ and 3′ sequences flanking the region in the C. albicans genome targeted for deletion.

The generation of homozygous deletion derivative mutants was carried out in a stepwise manner with the first successful transformation producing a heterozygous deletion derivative and the second successful transformation producing the desired homozygous deletion derivative. Transformations were performed as described by De Backer et al. (64). Transformed cells were selected for by growth on YPD agar medium containing 200 μg/ml nourseothricin (NAT), resistance to which was imparted by the ca*SAT1* gene. Incorporation of the deletion construct at the appropriate locus was verified by PCR amplification, as was the subsequent excision of the recyclable cassette from the genome. The recyclable flipper construct encodes a site-specific recombinase, ca*FLP*, under the control of a maltose-inducible promoter, allowing the selection cassette to be excised from the genome by growth of verified transformants in YP-maltose liquid medium.
