# Supplementary material for: Role of the WOR1 Promoter of Candida albicans in Opaque Commitment
Source: mBio. 2021 Sep 7;12(5):e02320-21. doi: 10.1128/mBio.02320-21 (PMC8546583; doi:10.1128/mBio.02320-21)
Supplement: FIG S1 [file mbio.02320-21-sf001.pdf]

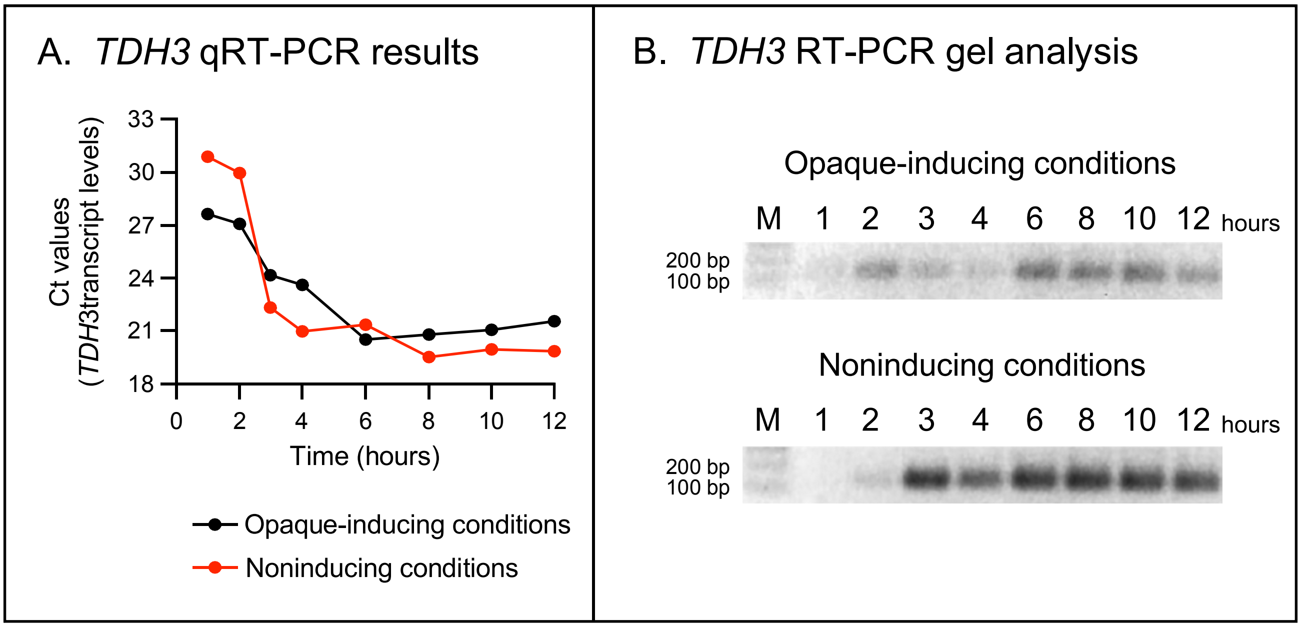

Supplemental Figure S1. Since the expression of housekeeping genes, commonly used as a reference to normalyze qRT-PCR analyses, can change over time as unbudded white cells exit stationary phase under noninducing or opaque-induced conditions, they could not be used to normalyze *WOR1* expression over time for qRT-PCR. Here we have analyzed the expression of *TDH3*, a gene commonly used in qRT-PCR analyses of *C. albicans*, to demonstrate this point. Values are presented in threshold cycles (Ct) in panel A and in RT-PCR gels in panel B. Because of such temporal changes and conditional variation in *TDH3* expression, and presumably other genes commonly used for data normalization, we have presented *WOR1* expression data using semi-quantitative RT-PCR in Figures 4, 7B and 9. Ct value, threshold cycle in PCR.

Supplemental Method for qRT-PCR analysis. RNA quality was confirmed to be higher than 9.0 RQI (RNA quality indicator) using the Experion RNA stdSens and highSens Analysis Kit (Bio-Rad). cDNA was generated from the purified RNA using the iScript cDNA synthesis kit (Bio-Rad). The amount of RNA used for generating cDNA was identical for samples from the opaque-inducing and noninducing conditions acquired at the same time point. However, because the quantity of RNA acquired at later time points was substantially greater than the quantity of RNA acquired for early time points (i.e., immediately after release from stationary phase), the amount of RNA used for generating cDNA varied between samples of the early and late time points. LightCycler 480 SYBR Green I Master mix (Roche) was used as indicated by the manufacturer in the qRT-PCR assay. The primer pair used can be found in Table S2.
