# Supplementary material for: Role of the WOR1 Promoter of Candida albicans in Opaque Commitment
Source: mBio. 2021 Sep 7;12(5):e02320-21. doi: 10.1128/mBio.02320-21 (PMC8546583; doi:10.1128/mBio.02320-21)
Supplement: TABLE S1 [file mbio.02320-21-st001.pdf]

Supplemental Table S1. Strains used in this study.

| Strain | Parent | genotype | Source |
|---|---|---|---|
| WO-1 | -- | *MTL*α/α blood stream isolate (IA, USA) | (1) |
| ΔP1 | WO-1 | *WOR1[p-wor1Δ(-7883 to -6850)::FRT]/WOR1[p-wor1Δ(-7883 to -6850)::FRT]* | This study |
| ΔP2 | WO-1 | *WOR1[p-wor1Δ(-6536 to -5090)::FRT]/WOR1[p-wor1Δ(-6536 to -5090)::FRT]* | This study |
| ΔP3 | WO-1 | *WOR1[p-wor1Δ(-4536 to -3213)::FRT]/WOR1[p-wor1Δ(-4536 to -3213)::FRT]* | This study |
| ΔP4 | WO-1 | *WOR1[p-wor1Δ(-2919 to -2138)::FRT]/WOR1[p-wor1Δ(-2919 to -2138)::FRT]* | This study |
| ΔP6 | WO-1 | *WOR1/[p-wor1Δ(+3050 to +3740)::FRT]/WOR1/[p-wor1Δ(+3050 to +3740)::FRT]* | This study |
| ΔP1-2 | WO-1 | *WOR1[p-wor1Δ(-7883 to -5090)::FRT]/WOR1[p-wor1Δ(-7883 to -5090)::FRT]* | This study |
| ΔP1-3 | WO-1 | *WOR1[p-wor1Δ(-7883 to -3213)::FRT]/WOR1[p-wor1Δ(-7883 to -3213)::FRT]* | This study |
| ΔP1-*wor1* | WO-1 | *WOR1[p-wor1Δ(-7883 to -)::FRT]/WOR1[p-wor1Δ(-7883 to +2438)::FRT]* | This study |
| P37005 | -- | *MTL***a**/**a** oral isolate from healthy individual (FL, USA) | (2) |
| PΔP1 | P37005 | *WOR1[p-wor1Δ(-7883 to -6850)::FRT]/WOR1[p-wor1Δ(-7883 to -6850)::FRT]* | This study |
| PΔP2 | P37005 | *WOR1[p-wor1Δ(-6536 to -5090)::FRT]/WOR1[p-wor1Δ(-6536 to -5090)::FRT]* | This study |
| PΔP3 | P37005 | *WOR1[p-wor1Δ(-4536 to -3213)::FRT]/WOR1[p-wor1Δ(-4536 to -3213)::FRT]* | This study |
| PΔP4 | P37005 | *WOR1[p-wor1Δ(-2919 to -2138)::FRT]/WOR1[p-wor1Δ(-2919 to -2138)::FRT]* | This study |
| PΔP6 | P37005 | *WOR1/[p-wor1Δ(+3050 to +3740)::FRT]/WOR1/[p-wor1Δ(+3050 to +3740)::FRT]* | This study |
| PΔP1-2 | P37005 | *WOR1[p-wor1Δ(-7883 to -5090)::FRT]/WOR1[p-wor1Δ(-7883 to -5090)::FRT]* | This study |
| PΔP1-3 | P37005 | *WOR1[p-wor1Δ(-7883 to -3213)::FRT]/WOR1[p-wor1Δ(-7883 to -3213)::FRT]* | This study |
| PΔP1-*wor1* | P37005 | *WOR1[p-wor1Δ(-7883 to -)::FRT]/WOR1[p-wor1Δ(-7883 to +2438)::FRT]* | This study |


1. Slutsky B, Staebell M, Anderson J, Risen L, Pfaller M, Soll DR. 1987. "White-opaque transition": a second high-frequency switching system in Candida albicans. J Bacteriol 169:189–197.
2. Lockhart SR, Pujol C, Daniels KJ, Miller MG, Johnson AD, Pfaller MA, Soll DR. 2002. In Candida albicans, white-opaque switchers are homozygous for mating type. Genetics 162:737–745.
