# Supplementary material for: Role of the WOR1 Promoter of Candida albicans in Opaque Commitment
Source: mBio. 2021 Sep 7;12(5):e02320-21. doi: 10.1128/mBio.02320-21 (PMC8546583; doi:10.1128/mBio.02320-21)
Supplement: TABLE S2 [file mbio.02320-21-st002.pdf]

Supplemental Table S2. Primers used in this study.

Primer	Purpose	Sequence
	Primers used for RT-PCR analysis	
THD3-f	*TDH3* RTPCR probe	ATGGGGTAAATCTGGTGTTGAC
TDH3-r	*TDH3* RTPCR probe	AGCAGATGGAGCAGTGATGATA
WOR1-f	*WOR1* RTPCR probe	GCGTAAATTTGGTCCCGACG
WOR1-r	*WOR1* RTPCR probe	CGCAAGCAACATTGGACCTG
	Primers used to generate *wor1* deletion cassettes	
5’ΔP1f	ΔP1, ΔP1-2, ΔP1-3, ΔP1-*wor1*	TACAACTAAGGTACCGTGTATATGATTGATCAC
5’ΔP1r	ΔP1, ΔP1-2, ΔP1-3, ΔP1-*wor1*	CCTTTTTTGGGCCCTCCTCTCTTGGTTGATC
3’ΔP1f	ΔP1	GATTACCTGTTGCGGCCGCTGGTGATCAATTTAATG
3’ΔP1r	ΔP1	CGAATTGAATGTCCCGCGGTGATATGCTTTATGCC
5’ΔP2f	ΔP2	CGATTTGTAAAAGGTACCAAATACGCTCC
5’ΔP2r	ΔP2	CAACCAAAACTCGAGTTCTAAAACTTACAATGTCC
3’ΔP2f	ΔP2, ΔP1-2	ATATGCGGCCGCCAGGAGAGAGCTGTAAACACTACAC
3’ΔP2r	ΔP2, ΔP1-2	GGTAGAGATTTGACATTTCCGCGGTACAAAGATA
5’ΔP3f	ΔP3	GTTAACAAAGAGGGGGCCCTATTATCAG
5’ΔP3r	ΔP3	GTATGGGTTTTCTCGAGGTAGGTAGGTAGTAGTG
3’ΔP3f	ΔP3, ΔP1-3	CAAAACAAGGACGCGGCCGCGGACGTGGACTC
3’ΔP3r	ΔP3, ΔP1-3	CCTTTATAGTTACAATGAGCTCTTGATTGCG
5’ΔP4f	ΔP4	GCATTCTAGTTGGGCCCCTTGCTACCAGTTTTC
5’ΔP4r	ΔP4	GAATTAAGCACCTCGAGCTGAATCTTGG
3’ΔP4f	ΔP4	CCACTGGGTGTAAAGCGGCCGCATTCGAATGTCAG
3’ΔP4r	ΔP4	AGGGATGCTAGAGCTCAATTGAATATGTGTTGTGCAG
5’ΔP6f	ΔP6	GAATCACATGGTACCTGGATGATCAATACTACTTAGAG
5’ΔP6r	ΔP6	ATATCTCGAGGTATGATGATTTTCTGGATTTCCGTG
3’ΔP6f	ΔP6	ATATGCGGCCGCTTCCTTAGAAAGAGATATAGAGATG
3’ΔP6r	ΔP6	AAATCCGCGGGTATGTGATGTTGTTTTG
3’wor1f	ΔP1-*wor1*	ATATGCGGCCGCGTGGGTCTGTGTGTGAATTCGTG
3’wor1r	ΔP1-*wor1*	TATACCGCGGCAATATTACAATTCCCTTCATG
